# Photoinitiated Polymerized Chitosan and DMDAAC for Efficient Algae Removal: Preparation, Characterization, and Application

**DOI:** 10.3390/polym18050556

**Published:** 2026-02-25

**Authors:** Tian Yang, Peng Zhang, Shanshan Dong, Liming Li

**Affiliations:** 1 College of Environment and Life Sciences, Shanxi University of Electronic Science and Technology, Linfen 041000, China; yt0916@163.com; 2 College of Environment and Ecology, Taiyuan University of Technology, Taiyuan 030024, China; dss1213812@163.com (S.D.); ob8yrk@163.com (L.L.)

**Keywords:** photoinitiation, chitosan, binary copolymerization, algae, flocculant

## Abstract

In this study, we used CTS and DMDAAC as raw materials and prepared a novel chitosan graft copolymer, CTS-g-PDMDAAC, through UV initiation in the presence of the photoinitiator VA-044. The synthesis process was systematically optimized, and its structural characteristics and performance in water treatment were evaluated. A single-factor experiment determined the optimal synthesis conditions to be a mass ratio of chitosan to DMDAAC of 1:4, total reactant concentration of 15.5%, ultraviolet light exposure for 5 h, and concentration of VA-044 of 0.2%. CTS-g-PDMDAAC demonstrated superior performance overall to CTS according to various characterization methods, such as FTIR, XPS, XRD, and BET. The coagulation experiment showed that at a dosage of 6.0 mg/L, the removal rates of residual turbidity and chlorophyll a reach 0.58 NTU and 99.37%, respectively, and the generated flocs have a dense structure and exhibit strong shear resistance. Finally, the flocculation mechanism was explored. Compared with traditional flocculants, CTS-g-PDMDAAC has the advantages of efficient algae removal, lower sludge production, no secondary pollution, and potential for the utilization of microalgae. This research provides theoretical support and suggests technical pathways for the development of biobased, environmentally friendly flocculants with broad pH adaptability.

## 1. Introduction

With the development of industry and agriculture, algal blooms caused by water eutrophication have become an increasingly serious issue, threatening the safety of drinking water and disrupting the ecological balance in water habitats. The demand for the utilization of algae as a resource further highlights the necessity of efficient separation technologies. The traditional physical method consumes a high amount of energy, the chemical oxidation method is prone to damaging the integrity of algal cells, and existing flocculants are associated with issues such as secondary pollution, extensive sludge production, and neurotoxicity. In contrast, chitosan-based flocculants are environmentally friendly and enable straightforward sludge treatment. Their applications in the field of environmental engineering are mainly concentrated in heavy-metal chelation, dye decolorization, and microalgae harvesting. However, their inherent defects urgently need to be overcome through modifications. At present, research on graft copolymerization modification of chitosan mostly focuses on thermal or chemical initiation systems, which are associated with problems such as high energy consumption, the presence of residual initiators, and degradation of molecular chains. The efficient removal of algae and protection of algal cell integrity with traditional coagulants are difficult to balance, which can easily lead to secondary pollution and hinder the utilization of microalgae.

Chitosan (CTS) is prepared through the deacetylation of chitin and is rich in active groups such as -NH_2_, -OH, and glycosidic bonds [1]. As a cationic flocculant, it can effectively remove suspended colloids and algae from water through electro-neutralization and bridging, and at the same time achieve microalgae recovery [2]. However, the natural properties of chitosan present significant bottlenecks: its limited molecular weight, insufficient cationic charge density, narrow dissolution range, and weak chemical stability limit its directly applicable pH range, thus making it difficult to meet the efficiency requirements of complex water quality issues [3]. In recent years, chemical modification has become a core strategy for circumventing chitosan’s performance bottleneck. Graft copolymerization significantly increases the molecular weight, charge density, and water solubility of chitosan by introducing cationic monomers into its main chain while retaining its biodegradability. In addition, quaternary ammonium modification enhances its electro-neutralization capabilities by introducing strongly positively charged groups, while carboxymethylation broadens its dissolution range by adding hydrophilic groups [4,5].

Based on this, the present study focused on the graft modification of chitosan (CTS) using dimethyldiallylammonium chloride (DMDAAC) as a cationic monomer. A novel flocculant, CTS-g-PDMDAAC (where PDMDAAC denotes the polymerized form of DMDAAC), was synthesized via a UV-initiated method. The objectives were to enhance its charge density, solubility, and thermal stability through synthesis optimization, thereby overcoming the pH sensitivity and efficacy limitations of conventional flocculants. This work aimed to provide a green and efficient solution for microalgae pollution control and resource utilization.

## 2. Materials and Methods

### 2.1. Materials

The reagents included chitosan (BR deacetylation degree 80–95%), dimethyldiallylammonium chloride (mass fraction 60%), azo diisobutylimidazoline hydrochloride (VA-044), azo diisobutylamidine hydrochloride (V50), Irgacure2959 and Irgacure1173 as initiators, glacial acetic acid, acetone, and anhydrous ethanol, all of analytical grade, and nitrogen with a purity of >99.99%.

The following instruments were used: magnetic stirrers (MS7-H550-S, DLAB Scientific Co., Ltd., Beijing, China), force-increasing stirrers (JJ-1, Guoyu Instrument Manufacturing Co., Ltd., Changzhou, China), ultraviolet lamps (300 W/365 nm, Xingchuang Electronic Co., Ltd., Guangzhou, China), electric-heating constant-temperature air-circulation-drying ovens (GZX-9140MBE, Boxun Industrial Co., Ltd., Shanghai, China), vacuum-drying ovens (BZF-50, Boxun Industrial Co., Ltd., Shanghai, China), electronic balances (FA2004B, Tianmei Balance Instrument Co., Ltd., Shanghai, China), pH meters (PHS-3C, INESA Instrument Co., Ltd., Shanghai, China), circulating water vacuum pumps (SHZ-D, Yuhua Instrument Manufacturing Co., Ltd., Zhengzhou, China), Zeta potential analyzers (Zetasizer Nano ZS90, Malvern Instruments Ltd., Malvern, UK), and infrared spectrometers (Nicolet iS10, Thermo Fisher Scientific, Waltham, MA, USA).

### 2.2. Synthesis of Flocculants

The chitosan was dried to a constant weight and dissolved in 2.0% glacial acetic acid solution by predetermined mass. Magnetic stirring was carried out until a homogeneous and transparent solution was formed [6,7]. The DMDAAC solution and photoinitiator were added, mixed well, filled with nitrogen, and deaerated for 20 min. The sealed reactor was exposed to ultraviolet light (wavelength of 365 nm) for 5 h, then left to stand for at least 4 h to purify the product.

**Figure 1:**
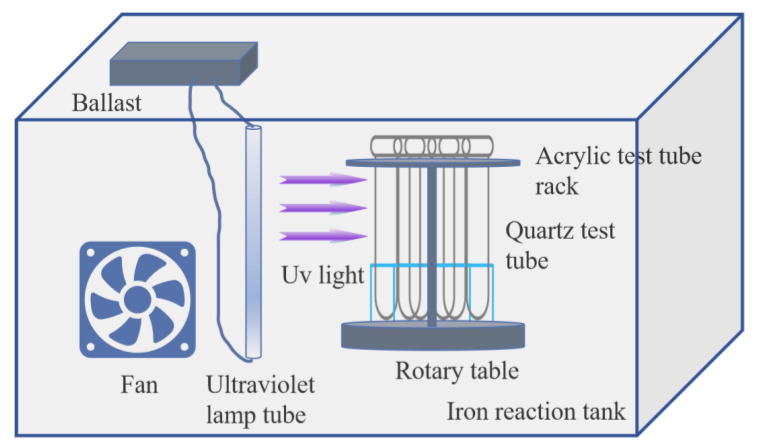
Synthetic experimental apparatus.

The synthesis device is shown in Figure 1.

### 2.3. Evaluation Indicators for Synthetic Effects

The grafting rate, as a key indicator for quantitatively characterizing the graft copolymerization efficiency, directly reflects the amount of cationic monomers grafted on each unit of chitosan in the system and represents the surface charge density of the final product. This parameter has a significant impact on the coagulation efficiency in the subsequent coagulation process, and is routinely determined by the gravimetric method. The formula used for its calculation is shown as Formula (1):

(1) $$G_{1}(\%)=\frac{W_{2}-W_{1}}{W_{1}}\times 100\%$$

In the formula,

G_1_ is the grafting rate;

W_1_ is the mass of chitosan, in g;

W_2_ is the mass of CTS-g-PDMDAAC, in g.

The utilization rate of dimethyldiallylammonium chloride is expressed by the mass conversion rate, and its calculation formula is as follows:

(2) $$G_{2}(\%)=\frac{W_{2}-W_{1}}{W_{3}}\times 100\%$$

In the formula,

G_2_ represents the mass conversion rate of DMDAAC;

W_3_ represents the mass of the added DMDAAC, in g.

### 2.4. Characterization Method of CTS-g-PDMDAAC

The detection methods and properties of CTS-g-PDMDAAC they measure are shown in Table 1.

### 2.5. Coagulation Experiment Steps

The kaolin was measured and dissolved in water. Then, the original algal solution was added in several portions to adjust the initial turbidity to 20.00–25.00 NTU and the chlorophyll a concentration to 0.5–1.0 mg/L. The flocculant was added and the coagulation was carried out by using a six-in-one stirring system according to its preset parameters. The settling time was 30 min. After static sedimentation, the residual turbidity of the supernatant and the concentration of chlorophyll a were measured to evaluate the flocculation effect.

### 2.6. Data Processing

All synthesis and coagulation experiments were independently repeated at least three times. Here, the experimental results are expressed as mean ± standard deviation. We used Origin 2021b software for data plotting and statistical analysis.

## 3. Results and Discussion

### 3.1. Factors Influencing Flocculant Synthesis

#### 3.1.1. The Influence of the Mass Ratio of CTS to DMDAAC on Branch Rate and Conversion Rate

**Figure 2:**
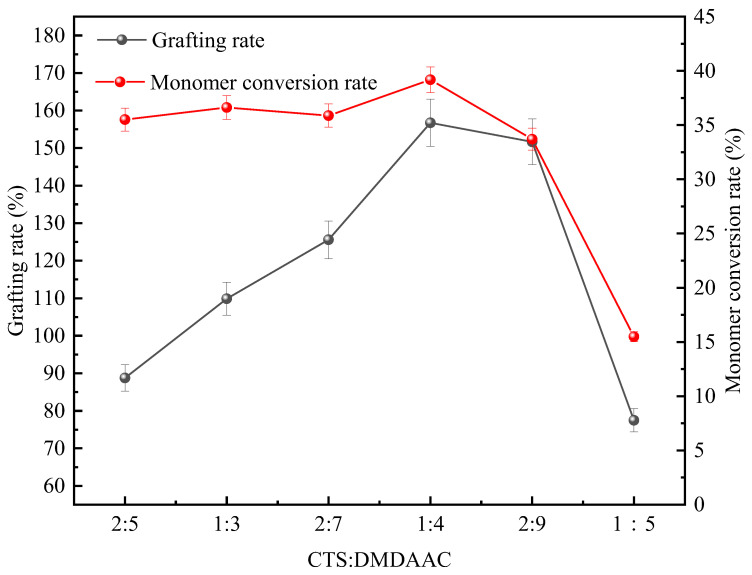
Effects of CTS-to-DMDAAC mass ratio on grafting rate and DMDAAC conversion rate.

The mass ratio of CTS (the main chain in the grafting reaction) to DMDAAC can directly affect the number of grafted monomers that each active site can receive, which impacts the grafting rate of the product and the conversion rate of DMDAAC. Meanwhile, if the proportion of CTS in the product is too high, this will reduce the solubility of the grafted product [9]. Figure 2 shows the changes in the grafting rate of the product and the conversion rate of DMDAAC when the reactant concentration is 15.5%, the light exposure time is 5 h, the photoinitiator concentration is 0.2%, and the mass ratios of CTS to DMDAAC are 2:9, 2:7, 2:5, 1:5, 1:4, and 1:3.

The experimental results show that with the increase in the proportion of DMDAAC, both the grafting rate of the product and the conversion rate of DMDAAC present a trend of increasing first and then decreasing. When m CTS/mDMDAAC = 1:4, both the grafting rate of the product and the conversion rate of DMDAAC reached their respective maximum values, which were 156.73% and 39.18%, respectively. This indicates that within a certain range, increasing the dosage of DMDAAC increases the grafting rate of the grafting product and the rate of DMDAAC conversion.

The graft copolymerization reaction site of CTS and DMDAAC is at the contact surface between the two. When the DMDAAC content is relatively low, an insufficient supply of monomers leads to the formation of a large number of unusable active sites on the surface of CTS, resulting in a significant decrease in the grafting rate [10]. Excessive CTS will lead to it exceeding its solubility limit in the reaction medium, resulting in the formation of CTS as undissolved solid particles or highly viscoelastic gel blocks. These undissolved components will significantly increase the viscosity of the system, limit the diffusion rate of monomers, and reduce the contact efficiency of active sites, ultimately resulting in a simultaneous decrease in both the grafting rate of the product and the rate of DMDAAC conversion [11]. However, as the specific gravity of DMDAAC increases, more graft monomers become available, while the chitosan content decreases, liquid viscosity drops, and particle movement accelerates. The combined effect of the two increases the probability of DMDAAC coming into contact with the active sites on CTS, allowing for sufficient contact and collision to occur during the polymerization reaction. The above reactions lead to the growth of the product chain, thereby increasing the grafting rate of the product and the rate of DMDAAC conversion. When the monomer concentration reaches its critical value, the content of CTS is insufficient to provide the effective active sites for copolymerization, which limits the number of monomers that can be grafted. It is difficult for the excess DMDAAC monomer to continue grafting due to the steric hindrance effect caused by the quaternary ammonium group carrying a large number of positive charges, resulting in a greater tendency for the monomer itself to undergo homopolymerization or cross-linking reactions. This is not conducive to the progress of the grafting reaction, thus resulting in a decrease in the yield of the target product [12]. To sum up, it is most appropriate to choose a mass ratio of CTS to DMDAAC of 1:4.

#### 3.1.2. The Influence of Total Reactant Concentration on Grafting Rate and Conversion Rate

**Figure 3:**
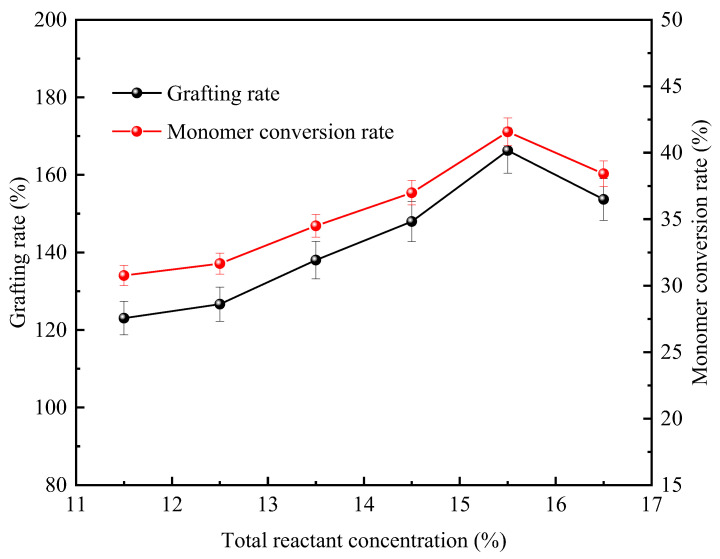
Effect of total reactant concentration on grafting rate and DMDAAC conversion.

The total reactant concentration is defined as the percentage of the total mass of reactants to the total mass of the solution. The total concentration of reactants can directly regulate the total amount of graftable monomers and the amount of chitosan that can effectively participate in the system. This parameter affects the final synthesis rate by changing the molecular collision frequency of reactants and the steric hindrance effect. Figure 3 shows the changes in the grafting rate of the product and the conversion rate of DMDAAC when the mass ratio of CTS to DMDAAC is 1:4, the concentration of the photoinitiator is 0.2%, the light exposure duration is 5 h, and the gradient of the total reactant concentration is set within a range of 11.5% to 16.5%, with a gradient difference of 1.0%.

The experimental results show that under constant reaction conditions, the total concentration of the system, the grafting rate, and the rate of DMDAAC conversion show a nonlinear pattern of first increasing and then decreasing. When the total concentration of the system reaches 15.5%, the grafting rate and grafting efficiency reach their peaks, which are 166.29% and 41.57%, respectively. In the early stage of the graft copolymerization reaction, the reaction degree is positively correlated with the concentration. This is because the increase in the concentration of reactants within the system enlarges the monomer diffusion area and enhances the effective contact probability, thereby promoting the improvement of the graft copolymerization reaction degree [13].

CTS still maintains a relatively high viscosity after being dissolved in a 2.0% glacial acetic acid solution. Moreover, DMDAAC is an oily liquid with a relatively high viscosity, and when its concentration exceeds 15.5%, the viscosity of the system increases sharply, the migration of free radicals is limited, and the solubility of chitosan decreases [14]. The above factors jointly inhibit the reaction process, reduce the reaction degree of the graft polymerization reaction, and result in a decrease in the grafting rate and monomer conversion efficiency. To sum up, the experiment shows that the optimal total concentration of the reaction system is 15.5%.

#### 3.1.3. The Influence of Reaction Time on Branch Rate and Conversion Rate

**Figure 4:**
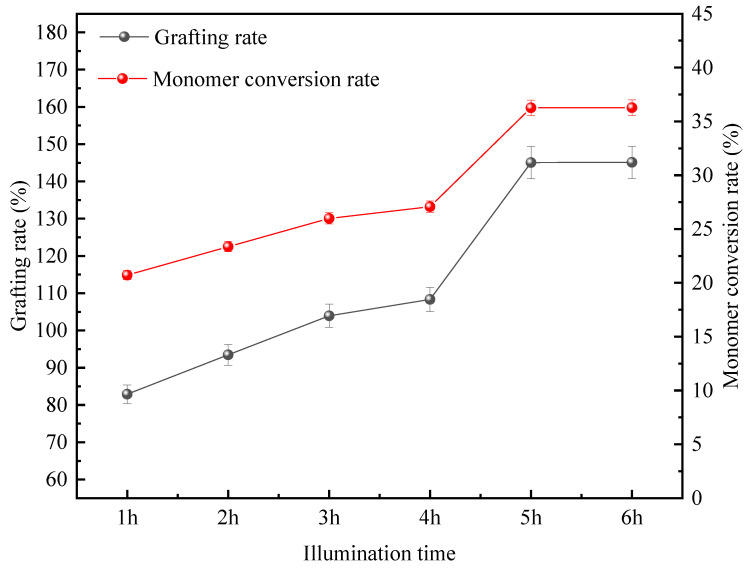
Effects of light duration on grafting rate and DMDAAC conversion rate.

The synthesis is initiated by ultraviolet light tubes with a power of 300 W. Both the duration and power of light exposure can affect the level of energy transferred. Therefore, the duration of light exposure also directly impacts the generation of branch products. Figure 4 shows the changes in the grafting rate of the product and the rate of DMDAAC conversion when the mass ratio of CTS to DMDAAC is 1:4, the concentration of reactants is 15.5%, and the concentration of photoinitiators is 0.2%. The gradient change in light exposure time is within the range of 1 h to 6 h, and the gradient difference is 1 h. It is important to note that this light exposure time is not the direct exposure time. The laboratory experimental setup is a test tube placed on a turntable and rotated at a constant speed (12 rpm); therefore, the actual direct exposure time is less than the experimental light exposure time.

The experimental results show that when the light exposure duration increases from 1 h to 5 h, the grafting rate of the product and the rate of DMDAAC conversion show a significant upward trend, reaching 145.05% and 36.26%, respectively, with 5 h of light exposure. After more than 5 h, both enter a growth plateau period, indicating that the photoinitiated polymerization reaction reaches equilibrium at this stage.

At the beginning of the light exposure stage, the reaction system appears pale yellow and transparent. Ultraviolet light passing through the liquid prompts the photoinitiator to generate free radicals, thereby significantly enhancing the collision efficiency of the active sites on the DMDAAC and CTS molecular chains and causing copolymerization [15]. As the exposure duration was extended to 5 h, the increased number of generated free radicals enabled the grafting rate and conversion rate to continuously rise to 145.05% and 36.26%, respectively. However, after more than 5 h, the formed product shows a tendency to cure. The increase in viscosity hinders the diffusion of monomers, which is not conducive to achieving contact between monomers and free radicals. The decrease in transparency makes it difficult for ultraviolet light to completely pass through the reaction system. The consumption of the initiator reduces the amount of free radicals formed and slows down the reaction rate. In addition, an excessively long reaction time is prone to causing homopolymerization of the monomer, and the grafting rate and the rate of DMDAAC conversion tend to stabilize [16]. In terms of the overall energy utilization efficiency, 5 h is determined as the optimal duration of illumination.

#### 3.1.4. The Influence of Initiator Concentration on Branching Rate and Conversion Rate

**Figure 5:**
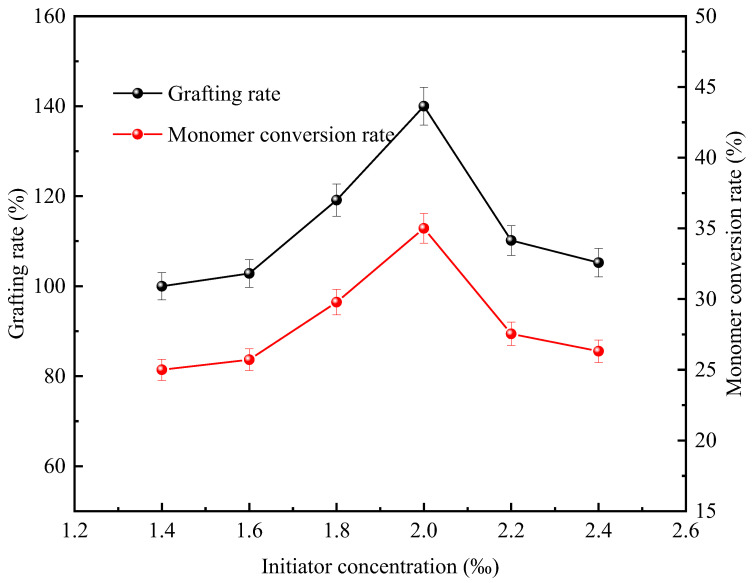
Effect of photoinitiator concentration on grafting rate and DMDAAC conversion rate.

As shown in Table 2, through previous experiments, it was concluded that VA-044 was the optimal photoinitiator out of V50, Irgacure2959, Irgacure1173, and VA-044. VA-044, as a water-soluble initiator, has the characteristics of excellent thermal stability, strong water solubility, and outstanding free radical homogeneity. Its molecular structure has the general formula R-N=N-R. The low bond energy characteristic of the R-N bond promotes thermal dissociation to generate free radicals, and then triggers the polymerization chain reaction. Our experiments have confirmed that the concentration gradient of the initiator significantly affects the synthesis of CTS-g-PDMDAAC. Figure 5 shows the changes in the grafting rate and the rate of DMDAAC conversion when the mass ratio of CTS to DMDAAC is 1:4, the reactant concentration is 15.5%, and the light exposure time is 5 h. The gradient change in the photoinitiator concentration is within the range of 0.14–0.24%, and the gradient difference is 0.02%.

The experimental data show that the initiator concentration has a significant effect on the efficiency of CTS-g-PDMDAAC synthesis. In the low concentration range, specifically, the range 0.0–0.2%, the grafting rate and monomer conversion rate show a significant positive correlation with the VA-044 concentration. When the dosage is 0.2%, the grafting rate is 140.00% and the conversion rate is 35.00%, both peaking simultaneously. After exceeding the critical concentration, both show a negative correlation, indicating that excessive initiator may trigger side reactions such as chain transfer, resulting in a decrease in the yield of the target product.

The reaction mechanism can be simply explained as the decomposition of VA-044 to form free radicals, which activate the active sites of CTS and drive the graft copolymerization reaction with DMDAAC. At a low concentration of VA-044, there are relatively few free radicals that can activate CTS. The insufficient density of active sites leads to a decrease in the probability of monomers making contact and thus limits the chain initiation efficiency. Therefore, the grafting rate of the product and the rate of DMDAAC conversion are relatively low. When the concentration increases to 1.5–0.2%, the increase in the density of activated free radicals leads to an increase in active sites, which promotes the chain growth rate, enabling the grafting rate and conversion rate to reach their peak [17,18]. When the concentration of VA-044 exceeds 0.2%, the high concentration of free radicals causes the number of active sites in CTS to exceed the number of monomers provided by DMDAAC, thereby inducing intermolecular self-polymerization of CTS. At the same time, the excessive free radicals trigger a double group termination reaction and the homopolymerization side reaction of DMDAAC, ultimately leading to a decrease in the grafting rate and monomer conversion rate [19]. To sum up, the results of this experiment indicated that 0.2% was the optimal initiator concentration.

### 3.2. Characterization of Flocculants

#### 3.2.1. FTIR

**Figure 6:**
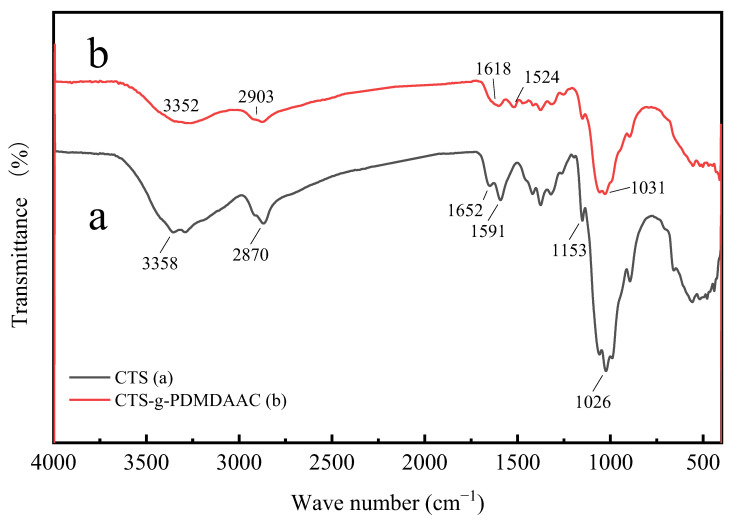
Fourier infrared spectra of CTS and CTS-g-PDMDAAC.

Infrared spectroscopy analysis was conducted on chitosan and its graft products, and the results are shown in Figure 6. By comparing lines (a) and (b), it can be seen that the two absorption spectra are similar, which indicates that the products of graft copolymerization have retained the chitosan groups and have a relatively small impact on the main chain of chitosan. It can be seen in Figure 6 (a) that the characteristic peaks of -OH and N-H in chitosan appear at 3358 cm^−1^, and the C-H stretching vibration peak of a primary alcohol appeared at 2870 cm^−1^. The characteristic peak at 1652 cm^−1^ is caused by the incomplete deacetylation of chitosan and the presence of acyl stretching vibration. The deformation vibration peak of N-H in -NH_2_ occurs at 1591 cm^−1^ [20,21]. In addition, the characteristic absorption peaks for CTS also include the stretching vibration peak of the asymmetric oxygen bridge bond (C-O-C) in the glycosidic bond at 1153 cm^−1^ and the characteristic peak of C-OH at 1026 cm^−1^ [22]. Compared with Figure 6 (a), in (b), the characteristic C=C peak of DMDAAC was located at 1618 cm^−1^ and the characteristic quaternary ammonium salt peak appeared at 1524 cm^−1^. This is because DMDAAC grafts to the -NH_2_ group in chitosan, causing its deformation [23]. Meanwhile, the numerous peaks that appeared within the range of 1500–1200 cm^−1^, displayed in Figure 6 (b), were caused by the deformation vibration of CH_3_ on CTS introduced by DMDAAC grafting, which introduced a large amount of quaternary ammonium groups [24]. In conclusion, by comparing the absorption spectra of the two, DMDAAC was successfully grafted onto the -NH_2_ group in chitosan while maintaining the integrity of the main chitosan chain.

#### 3.2.2. XRD

**Figure 7:**
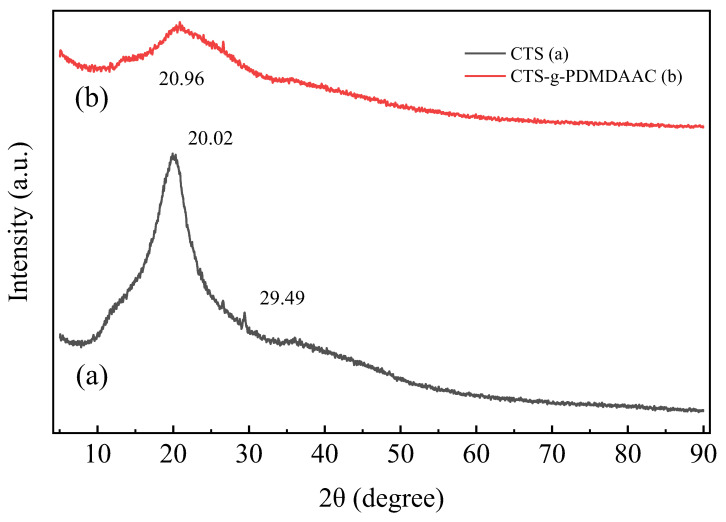
Diffraction spectra of CTS and CTS-g-PDMDAAC.

The differences in the crystal structures of CTS and CTS-g-PDMDAAC can be clearly observed through XRD diffraction spectroscopy characterization. As shown in Figure 7, by comparing the two, it is found that the XRD diffraction spectra of CTS have strong diffraction peaks at 2ϴ = 20.02° and 2ϴ = 29.49°, which correspond to the crystal form II of CTS [25,26]. This indicates that CTS has a crystal structure and exhibits strong intra- and intermolecular hydrogen bond interactions, which also explains its poor water solubility. In comparison, the diffraction spectrum of the grafted DMDAAC product CTS-g-PDMDAAC was relatively flat, showing an amorphous diffraction pattern. The diffraction peak at 2 ϴ = 20.96° does not correspond with the activity observed for CTS. This indicates that the original crystal structure of CTS has been disrupted. The hydrogen bonds decrease due to the consumption of a large amount of hydroxyl and amino groups via the connection of quaternary ammonium groups within DMDAAC, and the hydrogen bond effect weakens. This greatly enhances the hydrophilicity of CTS-g-PDMDAAC, making it readily soluble in water. Furthermore, after modification, the molecular structure of CTS-g-PDMDAAC becomes less organized and the amorphous regions increase. This is another factor that greatly enhances the solubility of the product in water with different pH values. In conclusion, by comparing the diffraction spectra of the two, it can be concluded that DMDAAC was successfully grafted onto chitosan, which changed the latter’s molecular structure and improved its solubility.

#### 3.2.3. BET

**Figure 8:**
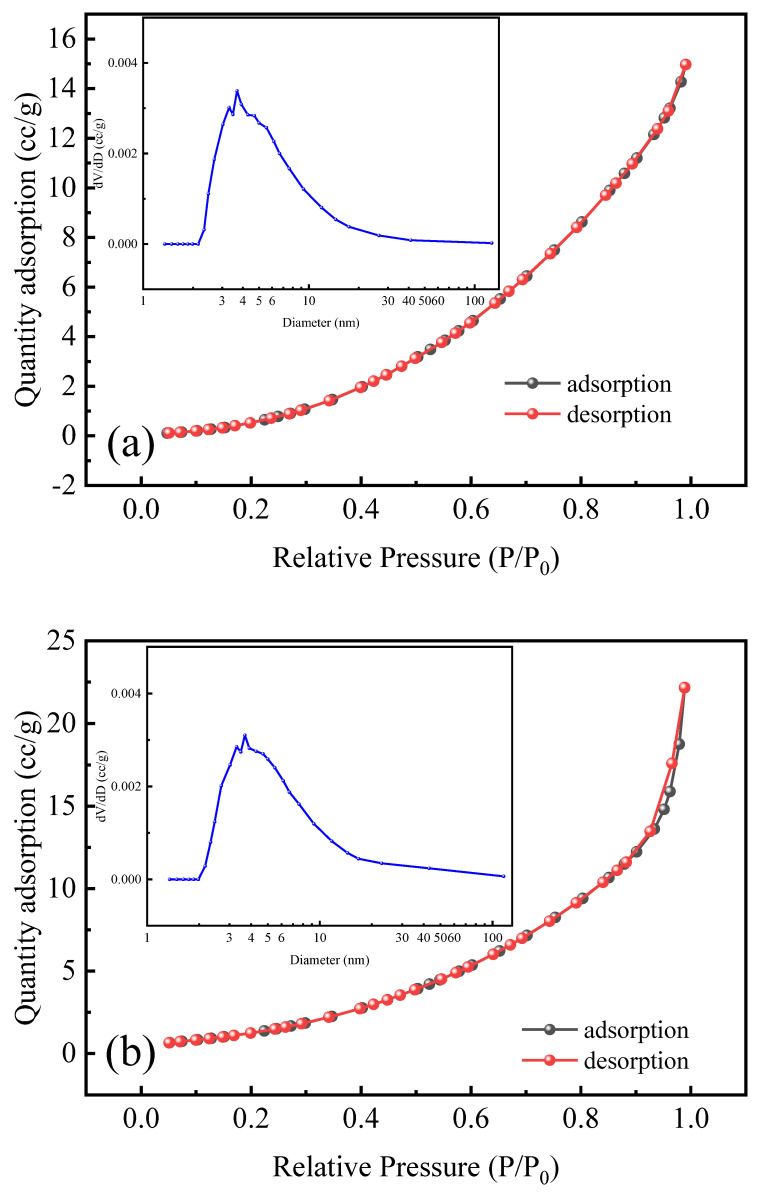
N_2_ adsorption–desorption isotherms and pore size distributions of CTS (**a**) and CTS-g-PDMDAAC (**b**).

Figure 8 shows the adsorption–desorption curves and pore size distribution of chitosan and CTS-g-PDMDAAC nitrogen. The adsorption–desorption curve of CTS nitrogen conforms to the characteristics of Class III isotherms, indicating that its surface is relatively smooth and the interaction with the adsorbed gas is weak. The CTS-g-PDMDAAC curve shows obvious hysteresis loops and is characteristic of a Class IV isotherm, indicating that it is a mesoporous material [27,28]. The pore size distributions of the two do not differ greatly. Most of the pores are distributed between 3.0 and 10.0 nm and are concentrated around 4.0 nm; thus, the material is classed as mesoporous. Table 3 shows the results of analyzing the other BET details of CTS and CTS-g-PDMDAAC. After graft copolymerization, the specific surface area, pore volume, and pore diameter of the copolymer CTS-g-PDMDAAC all increase, which provides a larger area and opportunity for contact with pollutants and, theoretically, leads to improved flocculation.

#### 3.2.4. SEM

**Figure 9:**
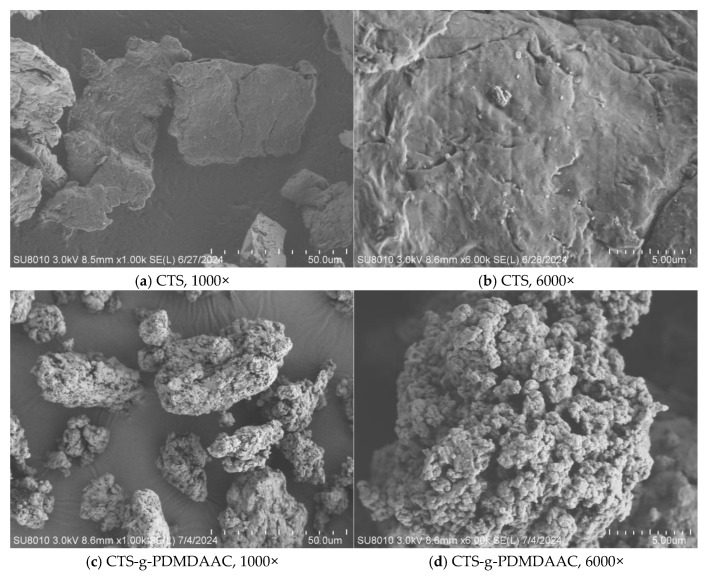
SEM images of CTS and CTS-g-PDMDAAC.

The scanning electron microscope images of CTS and CTS-g-PDMDAAC are shown in Figure 9. It can be seen in Figure 9a,c, showing CTS and CTS-g-PDMDAAC, respectively, both under 1000× magnification, that the surface of CTS is smooth, with fewer wrinkles and pores, presenting a crystal structure, while CTS-g-PDMDAAC has a rougher surface morphology and is loose and porous. In both images, under 6000× magnification, compared with the smooth surface of CTS, the surface of the copolymer is covered with pores of different sizes, and the typical crystal structure of CTS has disappeared. This is because the integration of DMDAAC weakens the hydrogen bonding of chitosan, thereby disrupting its structure. These well-developed pore structures not only increase the material’s specific surface area but also make it easier for it to form connections and bridges with pollutant particles, which is more conducive to the flocculation and removal of algal cells, and greatly improve its solubility.

#### 3.2.5. XPS

**Figure 10:**
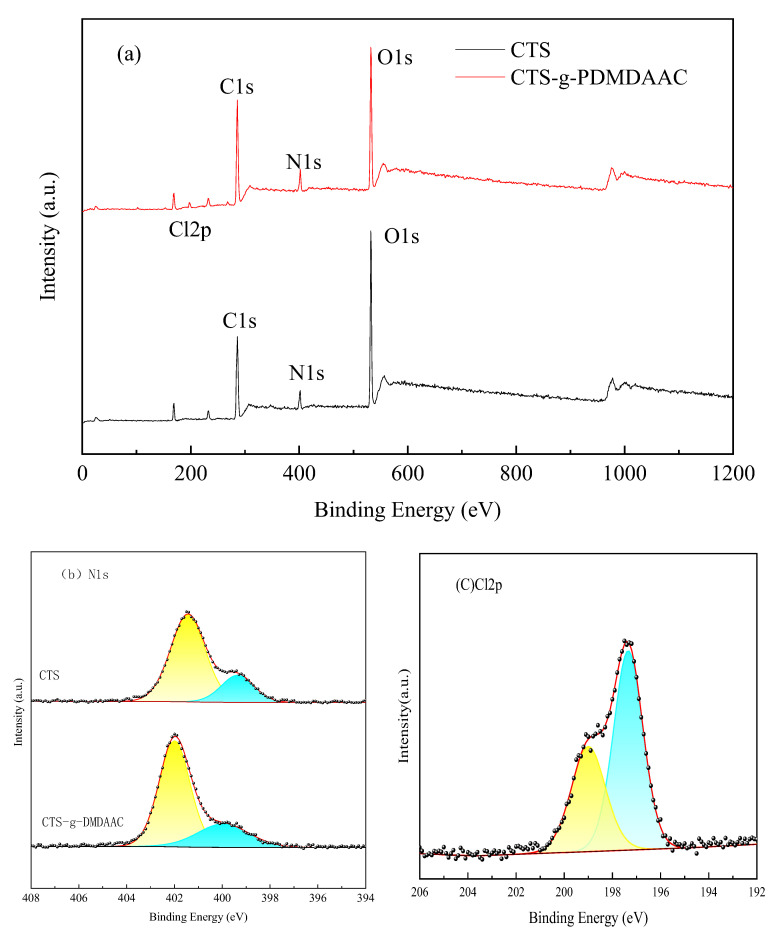
XPS spectra of CTS and CTS-g-PDMDAAC (**a**), N1s (**b**), and Cl2p (**c**).

The full spectra of chitosan and CTS-g-PDMDAAC were processed in software to detect peaks. It can be seen that the elemental peak of Cl2p appears in the CTS-g-PDMDAAC spectra at 198.0 eV in Figure 10a [29]. Meanwhile, Figure 10a also shows the changes in the relative content of elements before and after chitosan grafting. The relative content of O decreases and that of N increases.

In Figure 10b, both chitosan and its graft products can be separated into two peaks, namely, the amino functional group at 399.9 eV and C-N at 401.5 eV. By comparing the relative contents of both of their different functional groups, it can be concluded that the participation of amino groups in the reaction enables DMDAAC to be grafted onto the main chain of chitosan, reducing the original -NH_2_ peak [30]. Meanwhile, the access of DMDAAC adds a new quaternary ammonium nitrogen peak, -N^+^(CH_3_)_2_, which enhances the peak at 402.0 eV [31,32]. The coexistence of amino and quaternary ammonium nitrogen peaks indicates that direct addition of a quaternary ammonium to the main chain amino and PDMDAAC to the side link branches occurs simultaneously. As can be seen in Figure 10c, PDMDAAC contains Cl^−^ as a counterion. The appearance of Cl2p peaks with binding energies of approximately 197.0 eV and 199.0 eV, corresponding to 2p_3/2_ and 2p_1/2_ in Cl^−^, is an important indicator of successful grafting. The XPS analysis results indicate that DMDAAC is successfully grafted onto chitosan.

#### 3.2.6. Differential Thermal–Thermogravimetric Analysis

**Figure 11:**
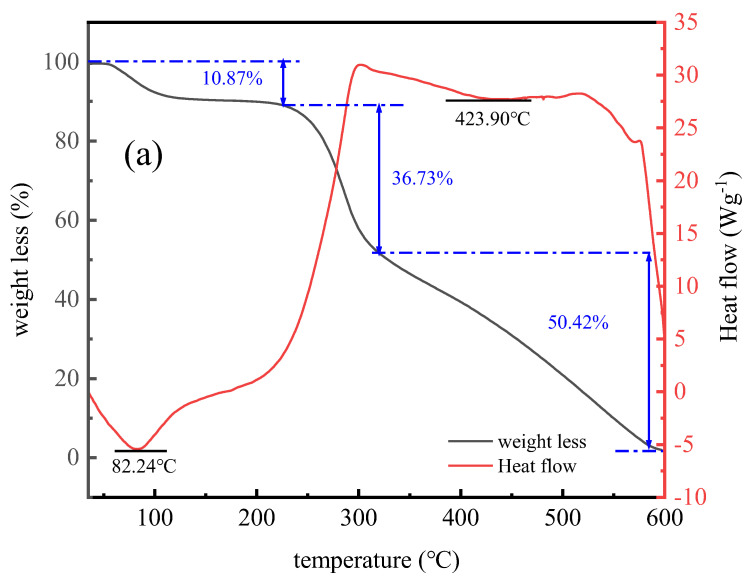

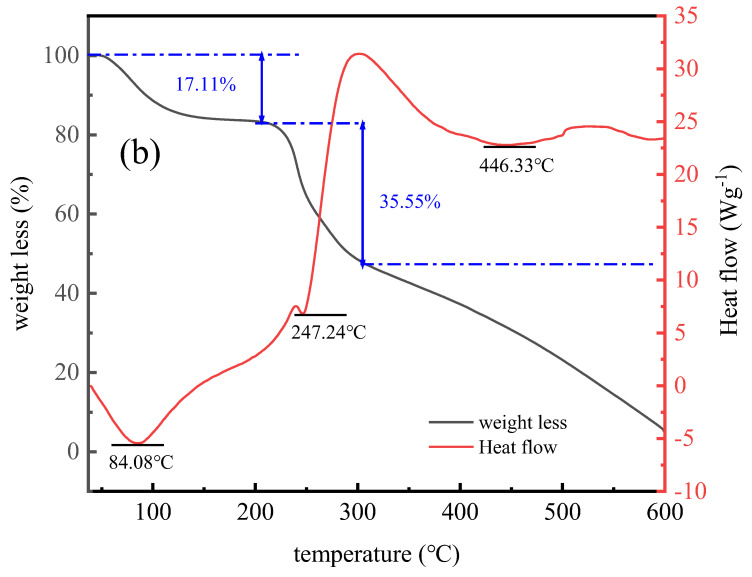
Differential thermogravimetric diagrams of CTS (**a**) and CTS-g-PDMDAAC (**b**).

The differential thermal and thermogravimetric analysis indicated that the thermal stability of CTS-g-PDMDAAC was significantly better than that of CTS. It can be deduced from Figure 11 that at a low-temperature (<200 °C), the CTS loses 10.87% of its weight due to water evaporation, while the weight lost by CTS-g-PDMDAAC reaches 17.11%. This is attributed to the weakened hydrogen bonds after grafting and the exposure of hydroxyl groups, resulting in an increase in the content of adsorbed and bound water. Correspondingly, in the DSC curve, two endothermic peaks for CTS and CTS-g-PDMDAAC occur at 82.24 °C and 84.08 °C, respectively, attributed to the evaporation of water. The quaternary ammonium groups in DMDAAC are hydrophilic and interact strongly with water molecules. They are difficult to detach from the quaternary ammonium groups and have a higher content of water molecules, which leads to a higher weight loss ratio and a higher endothermic peak temperature for CTS-g-PDMDAAC.

The weight of CTS decreases due to oligomer decomposition and main chain degradation at 200–330 °C and 330–600 °C, respectively (endothermic peak at 423.90 °C) [33]. In contrast, the second stage of weight loss (220–320 °C, 35.55%) for CTS-g-PDMDAAC originates from the methyl dissociation of the side chain of DMDAAC, with the endothermic peak of this stage occurring at 247.24 °C [34]. The third stage (320–600 °C) is dominated by the oxidation and decomposition of the main chain (endothermic peak at 446.33 °C).

To sum up, the similarities and differences between the differential thermogravimetric curves indicate that the decomposition temperature of the graft copolymerization product CTS-g-PDMDAAC is higher and the weight loss rate is lower, suggesting that the thermal stability of CTS-g-PDMDAAC is superior to that of CTS.

#### 3.2.7. Zeta Potential Analysis

**Figure 12:**
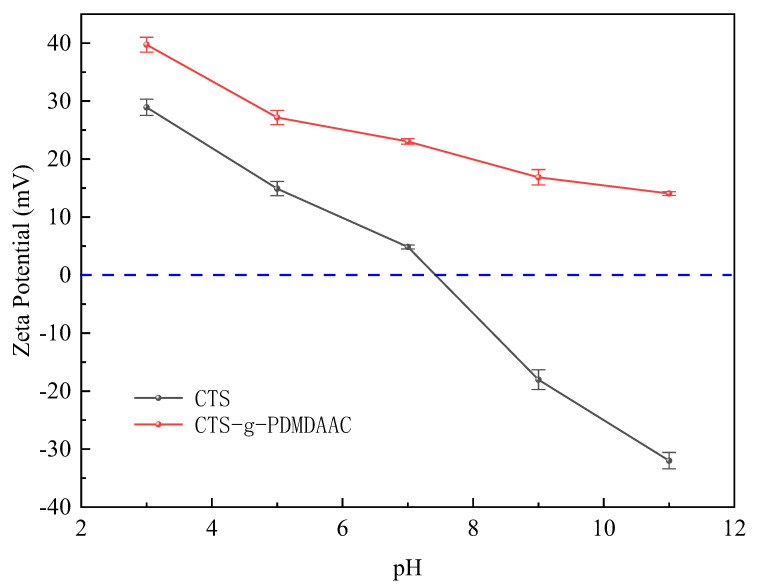
Variation in Zeta potential of CTS and CTS-g-PDMDAAC with pH.

The Zeta potential characterizes the charge characteristics of a particle’s surface. The inherent negative charge on the surface of algal cells makes them dependent on the charge-neutralizing ability of flocculants. As shown in Figure 12, CTS has a positive charge due to the protonation of -NH_2_ when pH < 7.0. However, as pH increases, this positive charge rapidly decays and becomes negative in weakly alkaline environments. In contrast, the Zeta potential of CTS-g-PDMDAAC is significantly higher than that of CTS under all pH conditions, and it maintains a positive charge under strongly alkaline conditions (pH = 11.0).

In acidic environments, the charge of CTS originates from amino protonation, and the positive charge density of CTS-g-PDMDAAC is significantly increased due to the dissociation of -N^+^(CH_3_)_3_ grafted into DMDAAC, promoted by H^+^ [35]. Under alkaline conditions, although OH^−^ partly neutralizes the positive charge, pH-independent ionization of the quaternary ammonium group enables CTS-g-PDMDAAC to remain positively charged, thus circumventing the pH sensitivity of CTS. The strong positive charge of CTS-g-PDMDAAC is attributed to the covalent grafting of quaternary ammonium groups, which endows it with stable electro-neutralization properties within a wide pH range and represents a key driving force for the efficient removal of negatively charged algal cells.

### 3.3. Application of Flocculants

#### 3.3.1. The Influence of Dosage on the Coagulation Effect

At room temperature, rapid stirring was set to 200 rpm/12 min and slow stirring was set to 50 rpm/20 min. The effects of CTS-g-PDMDAAC at a dosage of 2.0–12.0 mg/L and gradient of 2.0 mg/L on water with a high algae level and low turbidity was tested. The residual turbidity and chlorophyll a removal rate were used as evaluation indicators.

**Figure 13:**
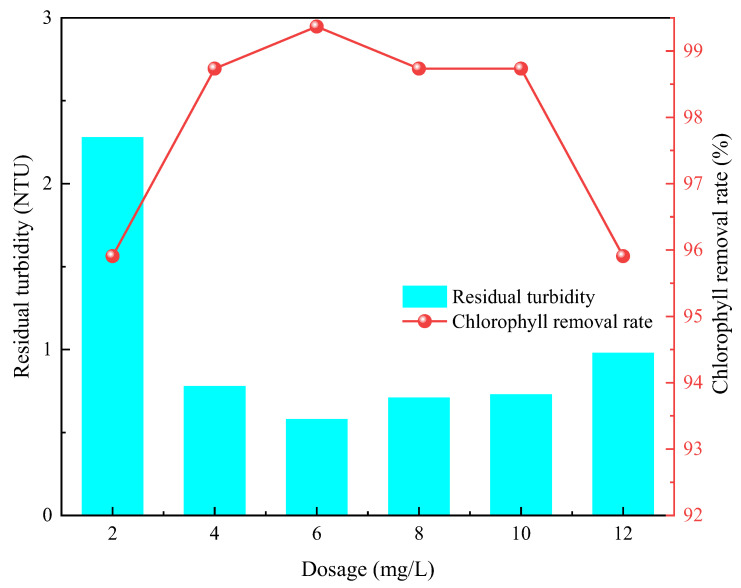
Influence of dosage on flocculation effect.

As shown in Figure 13, with the increase in dosage, the residual turbidity and chlorophyll removal rate show clear trends. The optimal dosage is 6.0 mg/L, the corresponding residual turbidity is 0.58 NTU, and the chlorophyll removal rate is 99.37%.

At a dosage of 2.0–6.0 mg/L, the proportion of CTS-g-PDMDAAC in the solution is too small, the charge neutralization is insufficient, the bridging effect is limited, and the flocculation efficiency is low. When the optimal dosage is reached, the charge neutralization is sufficient, a synergistic long-chain bridging effect is achieved, and colloid destabilization and floc growth reach a balance [36]. With continuously increasing dosage, the overload of positive charges on the surface of particles in the solution causes electrostatic repulsion, resulting in a “re-stabilization” phenomenon [37,38]. In contrast, it is also possible that the surface of the contaminated particles becomes coated by CTS-g-PDMDAAC, forming an adsorption film, which increases the steric hindrance between the two and causes floc dispersal. CTS-g-PDMDAAC achieves cost-effective optimization at 6.0 mg/L, providing an economical and efficient solution for the treatment of water with high levels of algae and low turbidity.

#### 3.3.2. Exploration of Flocculation Mechanism

##### Flocculation Analysis

**Figure 14:**
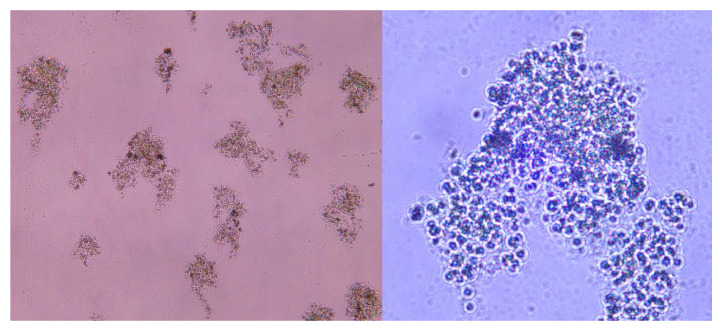
Optical micrograph of Chlorella flocs formed by CTS-g-PDMDAAC: (**left**) multiple flocs at low magnification (×100); (**Right**) Detailed morphology of a single floc, displaying complete algal cells (×400).

Microscopic observations (Figure 14) indicate that CTS-g-PDMDAAC forms large-sized flocs of algal cells through tight adsorption, with the morphology of the algal cells remaining intact without rupture due to stirring or mechanical action [39]. This effectively prevents the release of toxins within algal cells, eliminating the risk of secondary pollution. Simultaneously, it provides complete microalgal cells as a raw material for subsequent utilization, meeting the demands of green water treatment and the circular economy.

**Figure 15:**
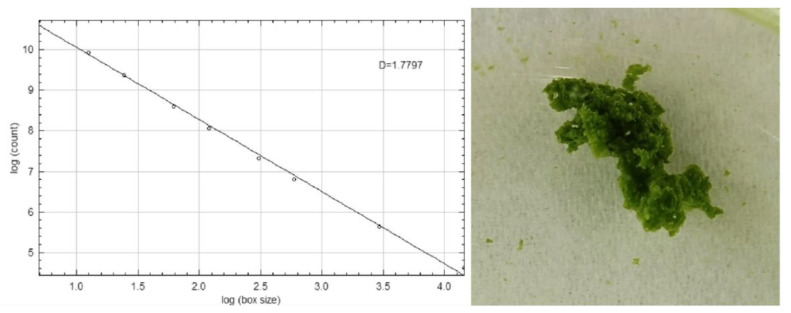
Fractal dimension analysis of Chlorella floc and magnification of floc.

After adjusting the flocculant dosage added to water with different levels of turbidity, there was a significant correlation between the fractal dimension of the flocs and the final turbidity of the treated water [40]. Observations under different experimental conditions show that when factors such as dosage, stirring conditions, and sedimentation time change, the structure of the flocs and, accordingly, the fractal dimension change [41]. Images were parsed and analyzed using the Image J software. Through calculating the box dimension, the two-dimensional fractal dimension of the algal flocs was obtained as 1.78, as shown in Figure 15. From this, it is proven that the sedimentation of the flocs produced after flocculation of CTS-g-PDMDAAC is improved.

##### Zeta Potential Analysis

The dominant mechanism of coagulation and its pathway of action were identified through the inherent correlation law between the coagulation effect and Zeta potential [42]. When the Zeta potential is negative, this often indicates that electrical neutralization plays a dominant role in the coagulation process. In contrast, when the potential turns positive, this indicates that other mechanisms of action, such as net capture and sweeping, have begun to exert their effects [43,44].

**Figure 16:**
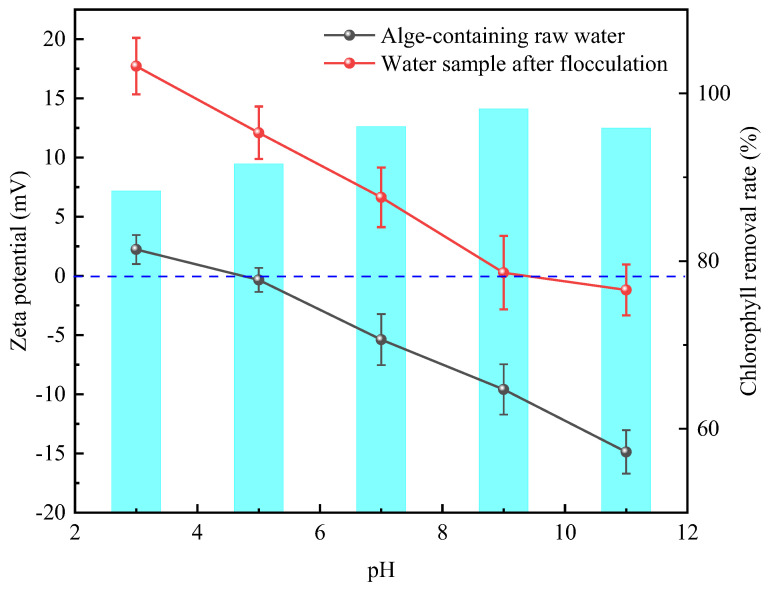
The relationship between the Zeta potential of the supernatant after CTS-g-PDMDAAC flocculation and the rate of chlorophyll-a removal at different pH values.

As shown in Figure 16, the electrochemical analysis indicates that the Zeta potential of the algae-containing raw water across a wide pH range is distributed near the isoelectric point or has a negative value range, which is consistent with the negative electric charge on the surface of algal cells. However, the Zeta potential of CTS-g-PDMDAAC is positive at pH 3.0–11.0, forming a significant charge gradient, driving the negatively charged algal cells toward directional adsorption via Coulomb attraction, and simultaneously compressing the double-layer structure of colloidal particles [45]. Therefore, it can be inferred that in the flocculation process, it is mainly electrical neutralization and compression of the double electric layer that play a role. As can be seen from Figure 16, when the Zeta potential of the solution is 0 mV, the electrostatic repulsion barrier—described by the DLVO theory—between particles is completely eliminated, the critical conditions for colloid stability are reached, and the flocculation efficiency reaches its peak [46,47]. This study confirmed that CTS-g-PDMDAAC can achieve efficient algae removal within the Zeta potential range of −8 to +2 mV (consistent with the optimal algae removal range reported by Henderson et al. [48]), and this is further enhanced at pH = 3.0 due to the superposition of bridging and net capture effects.

**Figure 17:**
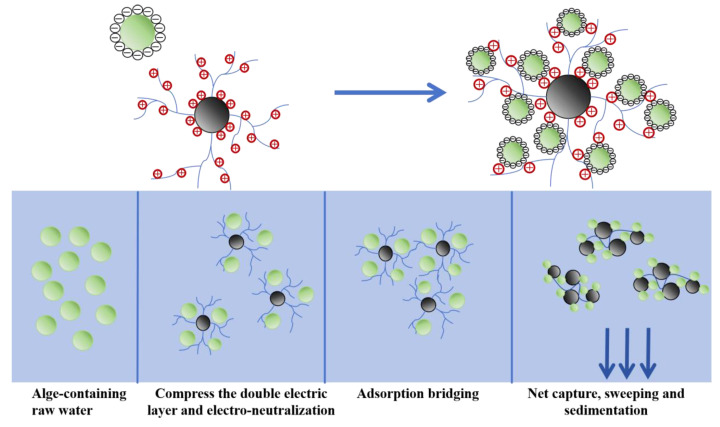
Flocculation removal process.

Based on the results of experiments investigating multiple dimensions, such as the characterization and analysis of CTS-g-PDMDAAC, the flocculation manifestations under various conditions, the Zeta potential changes during coagulation, and assessment of algal flocs, the coagulation and the algae removal process is shown in Figure 17. It can be deduced from this that CTS-g-PDMDAAC continuously compresses the surface diffusion layer of algal particles and exerts an electrical neutralization effect, causing the Zeta potential value on their surfaces to steadily increase, thereby promoting the rapid destabilization of the system and coagulation to form relatively tiny flocs. The CTS-g-PDMDAAC molecular chain promotes the gradual aggregation of tiny flocs to form larger and structurally stable flocs through bridging and high-specific-surface-area characteristics. During the sedimentation process, residual particles are effectively removed through the net capture and sweeping effect, achieving the coagulation and sedimentation of particles such as algal cells [49]. Ultimately, this has a purification effect on water bodies.

## 4. Conclusions

In this study, using VA-044 as a photoinitiator, a new chitosan-based graft copolymer, CTS-g-PDMDAAC, was successfully prepared through an ultraviolet light initiation method, and its synthesis, structural characteristics, and performance as a water treatment were systematically studied. The main conclusions are as follows:

(1) Synthesis optimization and innovation: Through single-factor experiments, the optimal synthesis conditions were determined as follows: A mass ratio of chitosan to DMDAAC of 1:4, a total reactant concentration of 15.5%, an ultraviolet light exposure duration of 5 h, and a concentration of initiator VA-044 of 0.2%. Under these conditions, the highest grafting rate of 166.29% and grafting efficiency of 41.57% were achieved, which were significantly superior to those reached using the traditional thermal initiation method. By precisely regulating the generation of free radicals, the ultraviolet light initiation strategy avoids the molecular chain degradation commonly seen in thermal initiation and simultaneously reduces energy consumption, providing a new route for large-scale production.

(2) Breakthroughs in structural functionalization and performance improvement: Infrared spectroscopy and XPS analysis jointly confirmed that DMDAAC was introduced into the CTS main chain through amino quaternization of the main chain and side link branches. The XRD analysis confirmed that its crystal structure was disrupted and presented amorphous characteristics, and the weakened hydrogen bonds enhanced hydrophilicity and solubility. The BET analysis showed that this provides more active sites for pollutant adsorption. SEM showed that the surface is rough and porous, and the thermogravimetric analysis confirmed the improvement of thermal stability. The Zeta potential indicates that the strong dissociation ability of the quaternary ammonium group endows the product with an excellent electro-neutralization capability, providing charge-driving for algal cell flocculation. Overall, CTS-g-PDMDAAC shows a much better overall performance compared to that of CTS.

(3) Advantages of flocculation performance and environmental significance: Through coagulation experiments, it is concluded that CTS-g-PDMDAAC presents obvious advantages in the field of water treatment. The results of analyzing the algal flocs show that this flocculant can prevent secondary pollution during water treatment, proving its effectiveness and practicability in this application, which is of great significance for ensuring the safety of water treatment and the utilization of microalgae as a resource. According to the the Zeta potential analysis, it can be concluded that the key mechanism of algae removal by CTS-g-PDMDAAC is predominantly electro-neutralization, supplemented with bridging and net capture effects, to achieve colloid destabilization and floc growth.

Aims for future work include revealing the structure–activity relationship between the conformation of graft chains and pollutant adsorption through molecular dynamics simulations, and comparing the full-cycle environmental costs of traditional flocculants. These efforts will facilitate the translation of chitosan-based materials from the laboratory to engineering applications, providing theoretical and technical support for the green transformation of water treatment technologies.

## Figures and Tables

**Table 1:** Physical and chemical property analysis table.

Characterization Method	Property Measured
FTIR [8]	Chemical and functional groups
Differential thermal–thermogravimetric analysis [8]	Thermal stability of substances
X-ray diffraction spectroscopy analysis [8]	Composition and form of crystalline substances
SEM [8]	Surface microscopic morphology
BET [8]	Specific surface area and aperture
Zeta potential analysis [8]	Surface electrical property and zero-point charge
XPS [8]	The composition, chemical state, and molecular structure of the surface elements of the material

**Table 2:** Comparison of primary grafting rates of various photoinitiators.

Types of Photoinitiators	V50	Irgacure2959	Irgacure1173	VA-044
Pre-selected grafting rate (%)	82.56%	52.38%	77.55%	103.81%

**Table 3:** CTS and CTS-g-PDMDAAC specific surface area, pore volume, and pore diameter.

Name	BET Specific Surface Area/(m^2^/g)	Hole Volume /(cc/g)	Aperture /(nm)
CTS	6.581	2.315	1.407
CTS-g-PDMDAAC	6.658	3.429	2.060

## Data Availability

The original contributions presented in this study are included in the article. Further inquiries can be directed to the corresponding authors.
